# A combination of flaxseed oil and astaxanthin alleviates atherosclerosis risk factors in high fat diet fed rats

**DOI:** 10.1186/1476-511X-13-63

**Published:** 2014-04-04

**Authors:** Jiqu Xu, Hui Gao, Li Zhang, Chang Chen, Wei Yang, Qianchun Deng, Qingde Huang, Fenghong Huang

**Affiliations:** 1 Department of Product Processing and Nutriology, OilCrops Research Institute, Chinese Academy of Agricultural Sciences, 2 Xudong Second Road, Wuhan 430062, P.R. China; 2 Hubei Key Laboratory of Lipid Chemistry and Nutrition, OilCrops Research Institute, Chinese Academy of Agricultural Sciences, 2 Xudong Second Road, Wuhan 430062, P.R. China; 3 Department of Nutrition and Food Hygiene, School of Public Health, Tongji Medical College, Huazhong University of Science and Technology, 13 Hangkong Road, Wuhan 430030, P.R. China; 4 Department of neurology, Hubei Xinhua Hosipital, 11 lingjiaohu Road, Wuhan 430015, P.R. China; 5 Department of Gastroenterology, The First People's Hospital of Yichang, The People's Hospital of China Three Gorges University, 2 Jiefang Road, Yichang 443000, P.R. China; 6 Department of Gastroenterology, The People's Hospital of China Three Gorges University, 2 Jiefang Road, Yichang 443000, P.R. China

**Keywords:** Flaxseed oil, Astaxanthin, Atherosclerosis, Oxidant stress, Plasma lipids, Inflammation

## Abstract

**Background:**

Atherosclerosis is the most common pathologic process underlying cardiovascular disease. Both flaxseed oil (FO) and astaxanthin (ASX) are believed to benefit cardiovascular system. The combined effect of FO and ASX on the atherosclerosis risk factors in rats fed a high-fat diet was investigated.

**Methods:**

Astaxanthin was dissolved in flaxseed oil to a final concentration of 1g/kg (FO + ASX). Male Sprague–Dawley rats were fed a rodent diet contained 20% fat whose source was lard (HFD) or 75% lard and 25% FO + ASX (50 mg ASX/kg diet) or 50% lard and 50% FO + ASX (100 mg ASX/kg diet) or FO + ASX (200 mg ASX/kg diet) for 10 weeks.

**Results:**

The combination of FO and ASX significantly increased the antioxidant defense capacity and decreased lipid peroxidation in plasma. Evident decreases in the levels TG, TC and LDL-C contents, as well as IL-6 and CRP were also observed in plasma of FO and ASX fed rats.

**Conclusion:**

The combination of FO and ASX can improve oxidative stress, lipid abnormalities and inflammation, providing evidence that the combination of FO and ASX could be a promising functional food in cardiovascular health promotion.

## Introduction

Nowadays, cardiovascular disease (CVD) is the leading cause of morbidity and mortality in most developed as well as many developing countries [1,2] and contributes substantially to healthcare budgets. Atherosclerosis is a chronic, progressive and systemic pathologic process and the primary contributing factor to CVD. Research into atherosclerosis has led to many compelling discoveries about the mechanism of the disease. There are definitive evidences to show that oxidant stress [3], lipid abnormalities [4] as well as chronic inflammation [5] have a crucial involvement in both the initiation and the progression of atherosclerosis.

Flaxseed oil (FO) is one of the most important specialty oils, which contains high levels of α-linolenic acid (ALA, 18:3 n-3). Higher intake of ALA has been long recognized as a “good nutritional intervention” with increasing many health benefits. As an essential polyunsaturated fatty acid (PUFA) that cannot be synthesized by human being, ALA serves as a precursor for long-chain n–3 polyunsaturated fatty acids such as eicosapentaenoic acid (EPA) and docosahexaenoic acid (DHA). Furthermore, ALA itself may exert various biological functions by competing with linoleic acid or interaction with ion channels and nuclear receptors [6]. ALA has been widely reported to have many beneficial effects on blood lipid profiles [6-9] and inflammation [6,10,11], which suggest that FO are beneficial for atherosclerosis prevention. However, on the other hand, since ALA is highly susceptible to oxidation, FO addition leads to a significantly higher tendency toward plasma lipid peroxidation [12,13], which may have an adverse effect on the protection of cardiovascular system.

Astaxanthin (ASX) is a lipophilic xanthophyll carotenoid and found in a variety of living organism including microalgae, fungi and crustaceans. It features a unique molecular structure which confers this natural product a powerful antioxidant activity [14]. In recent years, a large body of evidence has revealed a wide range of biological effects such as anti-cancer, anti-diabetes and neuroprotective actions [14,15]. In addition, ASX has also been reported to reduce blood pressure, LDL oxidation as well as inflammatory [14-17], and thus, combining its protection against oxidative stress, provides overall cardiovascular benefits.

Up to now, the effects of the combination of FO and ASX on cardiovascular system have not been investigated. In this study, we try to determine whether the combination of FO and ASX is able to reduce atherosclerosis risk factors in rats fed a high-fat diet.

## Materials and methods

### Chemical sources

The flaxseed oil was purchased from Caoyuankangshen Food Co., Ltd (Inner Mongolia, China). Astaxanthin extracted from microalga Haematococcus pluvialis was dissolved and diluted in flaxseed oil to a final concentration of 1g/kg (FO + ASX). Commercial deodorized lard was purchased from a local supermarket.

### Animals and diets

Forty male Sprague–Dawley rats (initially weighing 150–170 g) were purchased from Sino-British Sippr/BK (Shanghai, China). The animals were housed individually and maintained at a controlled ambient temperature (22 ± 1°C) under diurnal conditions (light–dark: 08:00–20:00) with access to laboratory chow and tap water ad libitum. After 1 week of acclimatization, rats were randomly divided into a high-fat diet (HFD) group and three experimental groups of 10 animals each. All animals were fed purified experimental diets which contained 35% maize starch, 20% casein, 15% sucrose, 5% cellulose, 3.5% mineral mixture (AIN-93M), 1% vitamin mixture (AIN-93M), 0.3% DL-methionine, 0.2% choline bitartrate and 20% fat. The fat in the diet of each group was provided by either lard (HFD group), or 75% lard and 25% FO + ASX (50 mg ASX/kg diet, L-FO + ASX group), or 50% lard and 50% FO + ASX (100 mg ASX/kg diet, M-FO + ASX group), or FO + ASX (200 mg ASX/kg diet, H-FO + ASX group). Every week, all ingredients for the purified diets were mixed, formed into a dough with purified water, rolled into pellets, sealed in air-tight plastic bags under nitrogen gas and stored at -80°C until use. The food in the animal cages was shaded from light and changed every day. The animals were cared for in accordance with *the Guiding Principles in the Care and Use of Animals*. The experiment was approved by the local animal care committee.

### Blood processing

After 10 weeks of feeding, all animals were fasted for 16 hours and killed under anaesthesia, blood was collected in heparinized vacutainer tubes from the heart immediately. Blood samples were centrifuged at 1500 g for 10 min at 4°C and the plasma was stored at -80°C until analysis.

### Plasma lipids analysis

The plasma triglyeride (TG), total cholesterol (TC), low-density lipoprotein cholesterol (LDL-C) and high-density lipoprotein cholesterol (HDL-C) levels were determined with commercial kits (Wako, Japan) by Hitachi 7020 full-automatic biochemical analyzer (Japan).

### Assay of plasma antioxidant capacity and lipid peroxidation

Superoxide dismutases (SOD) activity was measured according to the method of Kono [18]. Catalase (CAT) activity was estimated basing on the method of Goth [19]. Glutathione peroxidase (GPx) activity was measured by the method of Sazuka [20]. The glutathione (GSH) content was determined by the method of Moron [21]. The total antioxidant capability (T-AOC) was assayed with commercial kits (Nanjing Jiancheng Bioengineering Institute, China). Thiobarbituric acid reactive substances (TBARS) level was estimated by the method of Buege [22]. The detection procedure of these enzymes activities has been described in detail in our preceding report [23].

### Assay of plasma inflammatory markers

The plasma interleukin 6 (IL-6) and C-reactive protein (CRP) levels were measured by means of commercially available Rat CRP ELISA kit (Abcam, Cambridge, MA) and Rat IL-6 ELISA kit (Abcam, Cambridge, MA), respectively. All the procedures and conditions were consistent with the instructions of these kits.

### Statistical analyses

Values are presented as mean ± SEM (standard error of the mean). The data were analyzed by one-way ANOVA, followed by the Fisher PLSD post hoc test if the overall differences were significant (*p* < 0.05). All statistical analyses were performed using SPSS 13.0 statistical software (SPSS Inc., Chicago, IL) and a difference was considered significant when *p* < 0.05.

## Results

### Plasma antioxidative capacity and lipid peroxidation

**Figure 1:**
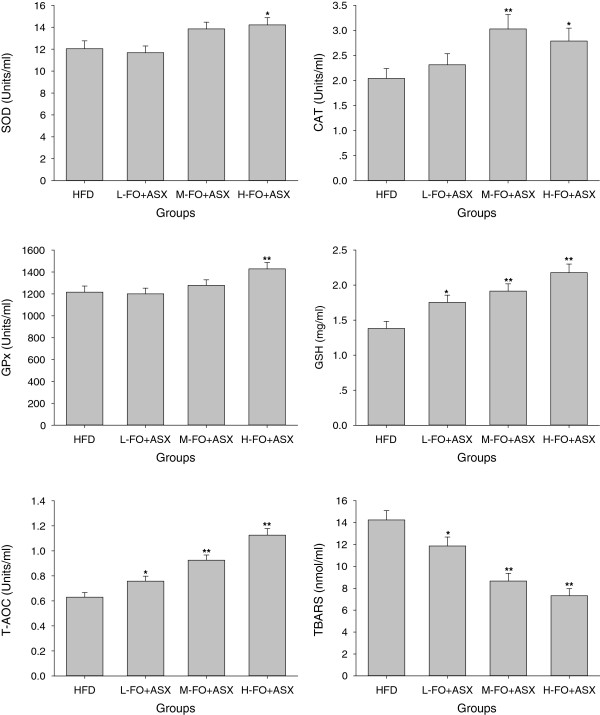
**Effects of FO and ASX combination on antioxidant enzymes (SOD, CAT and GPx) activities, GSH levels, T-AOC and TBARS contents in plasma of rats fed a high-fat diet.** HFD: high-fat diet group; L-. M- and H- FO + ASX: low, middle and high contents of FO and ASX combination groups. Bars represent the mean ± SEM from 10 animals in each group. * *p* < 0.05 and ** *p* < 0.01 compared to the HFD group.

H-FO + ASX-fed rats displayed significant SOD and GPx activities when compared with HFD-fed animals. CAT activities in M- and H-FO + ASX groups were remarkably higher than HFD group. Treatment with all doses of FO + ASX significantly increased GSH levels and total antioxidant capability. In addition, FO + ASX treatment also had substantially reduced content of TBARS relative to HFD diet (Figure 1).

### Plasma lipids

**Figure 2:**
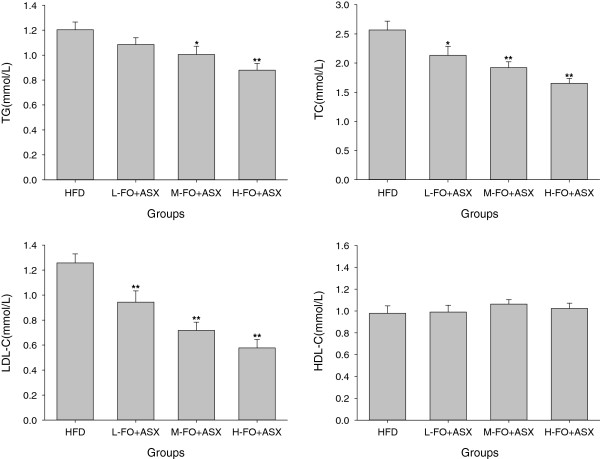
**Effects of FO and ASX combination on plasma TG, TC, LDL-C and HDL-C contents of rats fed a high-fat diet.** HFD: high-fat diet group; L-. M- and H- FO + ASX: low, middle and high contents of FO and ASX combination groups. Bars represent the mean ± SEM from 10 animals in each group. * *p* < 0.05 and ** *p* < 0.01 compared to the HFD group.

Figure 2 shows that replacement of the HFD with M- and H-FO + ASX significantly decreased plasma TG levels. Although HDL-C levels in plasma in all groups were comparable, rats administered with FO + ASX had markedly lower plasma TC and LDL-C levels than the HFD-fed animals.

### Plasma inflammatory

**Figure 3:**
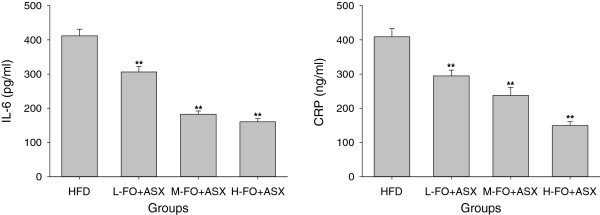
**Effects of FO and ASX combination on IL-6 and CRP levels in plasma of rats fed a high-fat diet.** HFD: high-fat diet group; L-. M- and H- FO + ASX: low, middle and high contents of FO and ASX combination groups. Bars represent the mean ± SEM from 10 animals in each group. ** *p* < 0.01 compared to the HFD group.

As seen in Figure 3, all three doses of FO + ASX treatment reduced plasma levels of IL-6 and CRP when compared with HFD diet.

## Discussion

Although the exact mechanisms remain to be delineated, oxidant stress [3], lipid abnormalities [4] as well as chronic inflammation [5] have been identified as the main trigger mechanisms of atherosclerosis. Atherosclerosis is fundamentally a metabolic disease subject to important dietary influences, and dietary lipids play a key role in the regulation of the development of atherosclerosis. High-fat diets, especially high-fat diet enriched with saturated fatty acid exert more deleterious effect on CVD and can lead to atherosclerosis [24], whereas the consumption of different kinds of fatty acids have various effects on atherosclerotic risk factors and even direct effects on atherogenesis [24-26]. Because of rich in ALA, FO has been proven to exert positive effect on atherosclerosis [25,26]. ASX is a potent natural antioxidant and may play a beneficial role in cardiovascular disease prevention [16]. In the study reported here, we evaluated the effect of FO and ASX combination on atherosclerosis risk factors in rats fed a high-fat diet.

Oxidative stress represents an imbalance between the free radical production and the antioxidant defense. The relative excessive production of free radicals, which can lead to oxidative damage to any biochemical component including lipids, proteins and DNA, plays a causative role in atherosclerosis [27]. For example, as a result of oxidative stress, LDL can be modified to oxidized LDL (oxLDL) which is clearly a critical factor in the atherosclerotic process and the cellular accumulation of oxidized LDL is considered a hallmark of atherosclerosis [28]. There is now consensus that oxidative stress is the pivotal pathogenetic factor and unifying mechanism for atherosclerosis and other cardiovascular diseases [29]. Therefore, an efficient antioxidant defense system is required to counteract the deleterious effects of oxidative stress. The primary antioxidant enzymes in mammals include SOD which converts superoxide to hydrogen peroxide, GPx and CAT which are responsible for converting hydrogen peroxide to water [30]. GSH is a very important non-enzymatic antioxidant which can react directly with free radicals or act as an electron donor in the reduction of peroxides catalyzed by GPx [31]. High consumption of dietary fat is a known cause of increased plasma oxidative stress [32], whereas in this experiment, the combination of FO and ASX remarkably elevated the plasma SOD, CAT and GPx activities as well as GSH level, which led to the pronounced enhancement of total antioxidant capability. As a result, lipid peroxidation levels in plasma markedly declined with the supplement of FO and ASX. However, FO itself is hardly thought to have an authoritative antioxidative activity and further, it may cause lipid peroxidation because of its susceptibility to oxidation [12,13]. Thus, ASX is anticipated to impart the entire antioxidative potency in this study. ASX has a unique chemical structure featured by the presence of polar moieties on both end of its polyene chain, and this structural property of ASX confers much greater free radical scavenging capability than β-carotene as well as α-tocopherol [33-35]. Besides, ASX is able to restore the activities of antioxidant enzymes SOD, CAT and GPx by inducing, at least in part, the Nrf2 pathway and other non-enzymatic antioxidants such as GSH, vitamins C and E in plasma and other various tissues in pathological conditions [36-38].

Hyperlipidemia is a well-known risk factor for arteriosclerosis as well as other cardiovascular disease and treatment of hyperlipidemia retards progression of arteriosclerosis [39]. There is mounting evidence that high-fat diet rich in saturated fatty acid leads to hyperlipidemia. However, in the present experiment, the levels of TG, TC and LDL-C in plasma declined in response to the consumption of FO and ASX combination and both of FO and ASX undoubtedly contributed to these beneficial changes. ALA has been shown to suppress the expression and activities of numerous hepatic fatty acid syntheses such as fatty acid synthase (FAS), malic enzyme and glucose 6-phosphate dehydrogenase [40,41], and hence decrease fatty acid synthesis in liver. On the other hand, ALA sharply enhances hepatic peroxisomal and mitochondrial fatty acid oxidation rate by increasing the expression and activities of a series of fatty acid oxidation enzymes [41,42]. As a peroxisome proliferator-activated receptor α (PPARα) agonist, ASX also shows the similar action on inducing fatty acid oxidation [36,43]. In addition, the hypocholesterolemic effects of FO and ASX are likely owing to elevated hepatic expression of LDL receptor [9,36] as well as declined cholesterol biosynthesis [43,44].

Contemporary advances in cardiovascular research have established a pivotal role for chronic inflammation in all stages of atherosclerosis [45-47]. Various proinflammatory risk factors such as oxLDL and infectious agents have a capability to trigger the production of proinflammatory cytokines which are deeply involved in the development and progression of atherosclerosis. As primary proinflammatory cytokines, IL-6 and CRP are sensitive measures of the burden of systemic atherosclerosis and extent of atherosclerotic activity [48-50]. In the present experiment, when the lard was replaced with the combination of FO and ASX, both the plasma levels of IL-6 and CRP collapsed, which implied that the FO and ASX combination is fully competent to improve inflammation status. Supporting our results, FO has been shown to suppress the expression of various inflammatory cytokines such as IL-6, IL-1, CRP and TNF-α via an activation of peroxisome proliferator-activated receptor γ (PPARγ) and/or a reduction in NF-κB induced gene expression [6,10,51]. Similarly, ASX also exerts antiinflammatory properties by suppressing NF-κB activation and thus decreased inflammatory markers levels in circulation [52,53].

In conclusion, supplement of FO and ASX combination has satisfactory efficacy at ameliorating oxidative stress, lipid profile and inflammation, which suggested that the combination of FO and ASX might contribute to prevent atherogenesis and then reduce the incidence of CVD. In addition, the presence of astaxanthin in FO lowers the lipid oxidation rate of FO and, on the other hand, astaxanthin is stable in FO in room temperature [54]. This makes the combination of FO and ASX very promising functional food in cardiovascular health promotion.

## Competing interest

No competing financial interests exist.

## Authors’ contributions

Author JX designed and wrote a first draft of the paper. HG, LZ, CC, WY and QD carried out all the experiments. QH performed the data analysis and created the figures. FH contributed to the design of the study, reviewed the manuscript and contributed to the final version. All authors contributed to and have approved the final manuscript.
